# Community-Acquired Pneumonia Caused by Avian *Chlamydia abortus*, the Netherlands

**DOI:** 10.3201/eid3103.241406

**Published:** 2025-03

**Authors:** Jairo Gooskens, Einar H.R. van Essen, Margriet E.M. Kraakman, Patrick Wörz, Edou R. Heddema, Stefan A. Boers

**Affiliations:** Center of Infectious Diseases (LUCID), Leiden University Medical Center, Leiden, the Netherlands (J. Gooskens, M.E.M. Kraakman, P. Wörz, S.A. Boers); Leiden University Medical Center Intensive Care, Leiden (E.H.R. van Essen); Reference Laboratory for Zoonotic *Chlamydia* Infections, Zuyderland Medical Center, Sittard-Geleen, the Netherlands (E.R. Heddema)

**Keywords:** pneumonia, chlamydia, metagenomics, zoonoses, Chlamydia abortus, respiratory infections, bacteria, the Netherlands

## Abstract

We report avian *Chlamydia abortus* pneumonia in an immunocompetent elderly patient in the Netherlands after environmental exposure to wild aquatic birds, including seabirds. New molecular surveillance studies are needed in wild and captive birds, as well as increased awareness to establish occurrence, clinical manifestations, and geographic distribution of this rare zoonotic disease.

The bacterial genus *Chlamydia* consists of 14 ubiquitous species that affect a wide range of hosts. The species *C. trachomatis*, *C. pneumoniae*, *C. psittaci*, *C. caviae*, *C. felis*, and *C. abortus* are pathogenic to humans after interhuman or zoonotic transmission. Taxonomy studies have identified a new avian *C. abortus* subgroup that is an intermediary related to *C. psittaci* and mammalian *C. abortus* (*1*). In 2024, avian *C. abortus* was associated with human respiratory tract infection and possible human-to-human transmission (*2*). We report a case of severe community-acquired pneumonia caused by an avian *C. abortus* genotype not yet associated with human disease.

A previously healthy 74-year-old man from a residential coastal town in the Netherlands was admitted to the hospital during the winter in 2021 with fever, confusion, and progressive dyspnea of 4-day duration. The patient was a nonsmoker and was vaccinated against seasonal influenza and SARS-CoV-2. He lived a socially withdrawn lifestyle and had no exposure to ruminants or domestic birds, although he regularly fed wild aquatic birds during the winter months. He reported noteworthy exposure to wild birds, including seabirds, which included hand feeding and occasional contact with bird droppings on his clothing. 

At hospital admission, a physical examination revealed a body temperature of 39.3°C, pulse of 162 beats/min, blood pressure of 127/77 mm Hg, and respiration rate of 42 breaths/min. Laboratory results showed an unremarkable leukocyte count of 7.49 × 10^9^ cells/L (reference range 4.0–10.0 × 10^9^ cells/L), acute lymphocytopenia of 0.43 × 10^9^ cells/L (reference range 1.0–3.5 × 10^9^ cells/L), unremarkable serum creatinine level of 101 µmol/L (reference range 64–104 µmol/L), and elevated C-reactive protein level of 305 mg/L (reference range <5.0 mg/L). Other findings included hyponatremia with a serum sodium level of 129 mmol/L (reference range 136–145 mmol/L) and elevated levels of creatine kinase, 531 U/L (reference range <171 U/L); plasma fibrinogen, 7.3 g/L (reference range 2.1–3.8 g/L); serum lactate dehydrogenase, 397 U/L (reference range <248 U/L); blood urea, 11.7 mmol/L (reference range 2.5–7.5 mmol/L); serum glucose, 10 mmol/L (reference range 3.9–7.7 mmol/L); γ-glutamyl transferase, 71 U/L (reference range <55 U/L); aspartate aminotransferase, of 87 U/L (reference range <35 U/L); and cardiac troponin T, 58 ng/L (reference range <14 ng/L). All other laboratory findings were unremarkable. 

The patient was transferred to the intensive care unit because of hypoxemic respiratory failure (SaO_2_ <90%) and progressive pulmonary consolidations with pleural effusion (Figure 1) requiring invasive mechanical ventilation. Blood and sputum cultures collected before intravenous cefuroxime and ciprofloxacin empirical therapy showed no microbial pathogens. Bronchial aspirate obtained by fiberoptic bronchoscopy was negative for respiratory viruses, *Legionella* spp., *Mycoplasma pneumoniae*, and *Chlamydia pneumoniae* by real-time PCR; however, B-CAP real-time PCR (Biolegio, https://www.biolegio.com) detected *Chlamydia* DNA. Quantitative 16S micelle PCR and next-generation sequencing–based (*3*) and metagenomic-based (*4*,*5*) microbiota profiling showed a high relative abundance of *Chlamydia* spp. in the lower respiratory tract; levels were >100 million 16S rRNA gene copies per milliliter of bronchial aspirate (Appendix Figure 1). Metagenomic profiling suggested a high abundance of *Chlamydia* spp. intermediaries related to *C. psittaci* and *C. abortus*. The reference laboratory detected *C. abortus* carrying a plasmid inherent in avian *C. abortus* (*6*). Multilocus sequence typing revealed sequence type 359. Phylogenetic analyses of concatenated multilocus sequence typing and plasmid II *xerC* gene sequences confirmed avian *C. abortus* (Figure 2) (*6*,*7*). Chlamydial outer membrane protein A gene sequencing identified a single *Chlamydia* spp. matching provisional genotype R54 (Appendix Figure 2), previously isolated from migratory seabirds in polar regions but never from humans or other mammals (*8*,*9*). The avian *C. abortus* clinical isolate KML-2021 (GenBank accession nos. OR665720 and PQ001575) obtained from the patient in this study was genetically different from avian *C. abortus* clinical strain NL2335-4C (GenBank accession nos. CP158097 and CP158098) and related clinical isolates or strains from other patients in the Netherlands (Figure 2; Appendix Figure 2) (*2*). 

**Figure 1:**
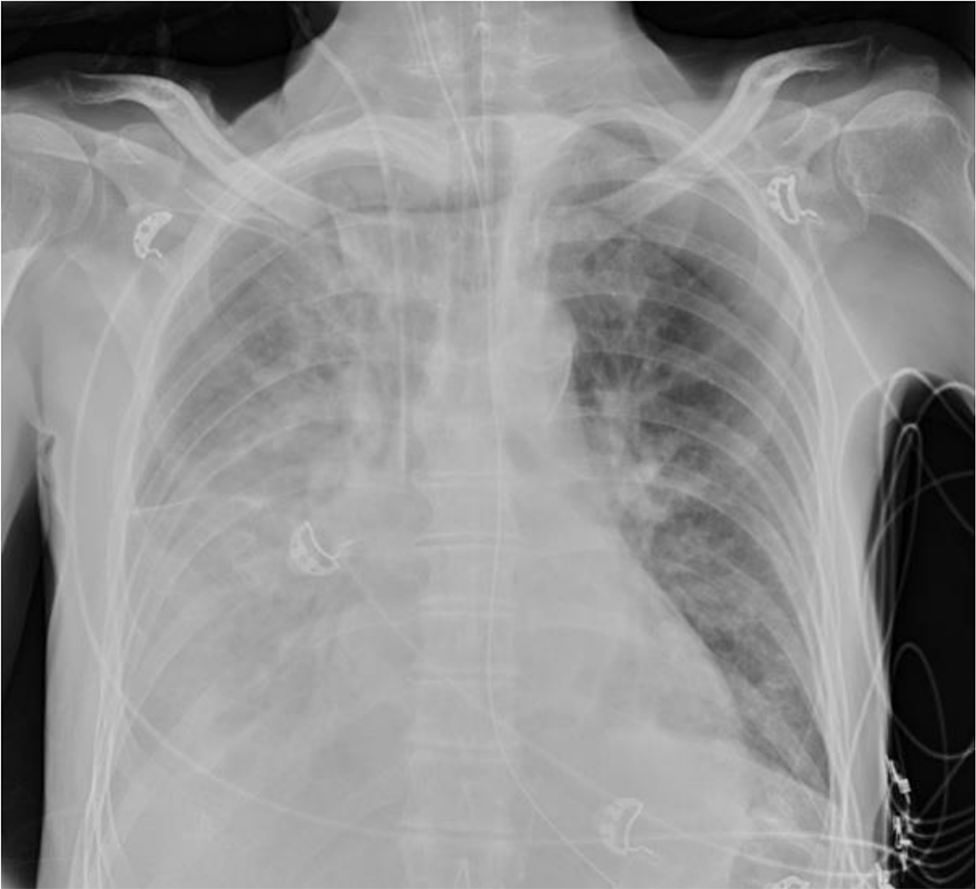
Chest radiograph of a patient with community-acquired pneumonia caused by avian *Chlamydia abortus*, the Netherlands. The radiograph shows progressive pulmonary consolidations with pleural effusion.

**Figure 2:**
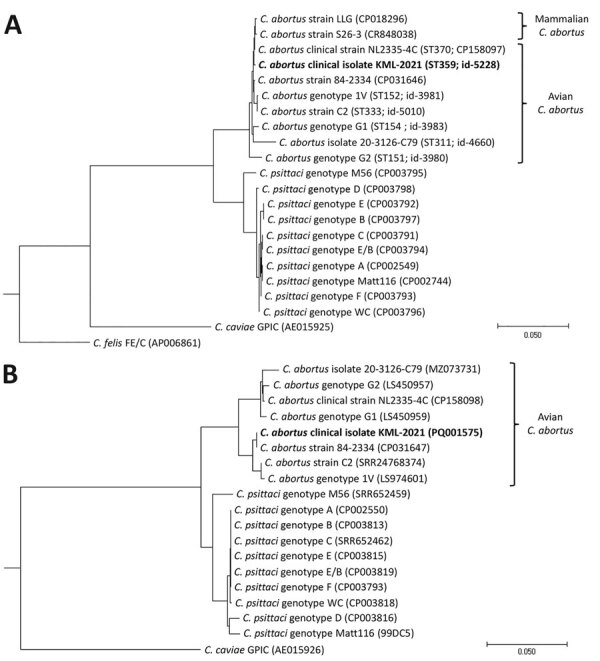
Chlamydial phylogeny of isolates from a patient with community-acquired pneumonia caused by avian *Chlamydia abortus*, the Netherlands, and reference sequences. Chlamydial phylogenetic trees were constructed by using concatenated MLST *gatA, oppA, hflX, gidA, enoA, hemN,* and *fumC* gene sequences (A) or by using plasmid II *xerC* gene sequences (B) of clinical isolate KML-2021 (bold; PubMLST sequence type 359; id-5228, https://pubmlst.org) and reference *Chlamydia* isolates (GenBank accession no. or PubMLST id shown) that were aligned and analyzed in MEGA11 (https://www.megasoftware.net). The phylogenetic tree was constructed by using maximum-likelihood approximation with FastTree v2.1.11 (https://kbase.us/applist/apps/kb_fasttree/run_FastTree/release) and rooted with *C. felis* (FE/C) or *C. caviae* (GPIC reference strains). Scale bar indicates nucleotide substitutions per 100 sites. id, identification; MLST, multilocus sequence typing.

The patient’s respiratory condition improved with doxycycline treatment directed at *Chlamydia* spp. At 1-year clinical follow-up, the patient noted no recurrences. The patient provided written informed consent for publication to the treating physician (E.H.R.v.E.) after the 1-year clinical follow-up. All authors confirmed that subject protection guidelines and regulations were strictly followed. We notified the Public Health Service for the Hollands Midden region in Leiden, the Netherlands, of the zoonotic *Chlamydia* spp. infection and did not identify any human-to-human transmission events.

In conclusion, our findings confirm the zoonotic potential of avian *C. abortus* to cause severe community-acquired pneumonia in humans. Increased awareness is warranted to establish the occurrence, clinical manifestations, and global geographic distribution of that rare zoonotic disease. We recommend molecular surveillance studies in wild and captive birds to evaluate sources of contamination of different avian *C. abortus* genotypes.

Appendix Bacterial microbiota profiles and chlamydial genotyping in a patient with community-acquired pneumonia caused by avian *Chlamydia abortus*, the Netherlands.
