# Supplementary material for: Community-Acquired Pneumonia Caused by Avian Chlamydia abortus, the Netherlands
Source: Emerg Infect Dis. 2025 Mar;31(3):630–3. doi: 10.3201/eid3103.241406 (PMC11878300; doi:10.3201/eid3103.241406)
Supplement: Appendix — Bacterial microbiota profiles and chlamydial genotyping in a patient with community-acquired pneumonia caused by avian Chlamydia abortus, the Netherlands. [file 24-1406-Techapp-s1.pdf]

Article DOI: https://doi.org/10.3201/eid3103.241406

EID cannot ensure accessibility for supplementary materials supplied by authors. Readers who have difficulty accessing supplementary content should contact the authors for assistance.

Community-Acquired Pneumonia Caused by Avian *Chlamydia abortus*, the Netherlands

Appendix

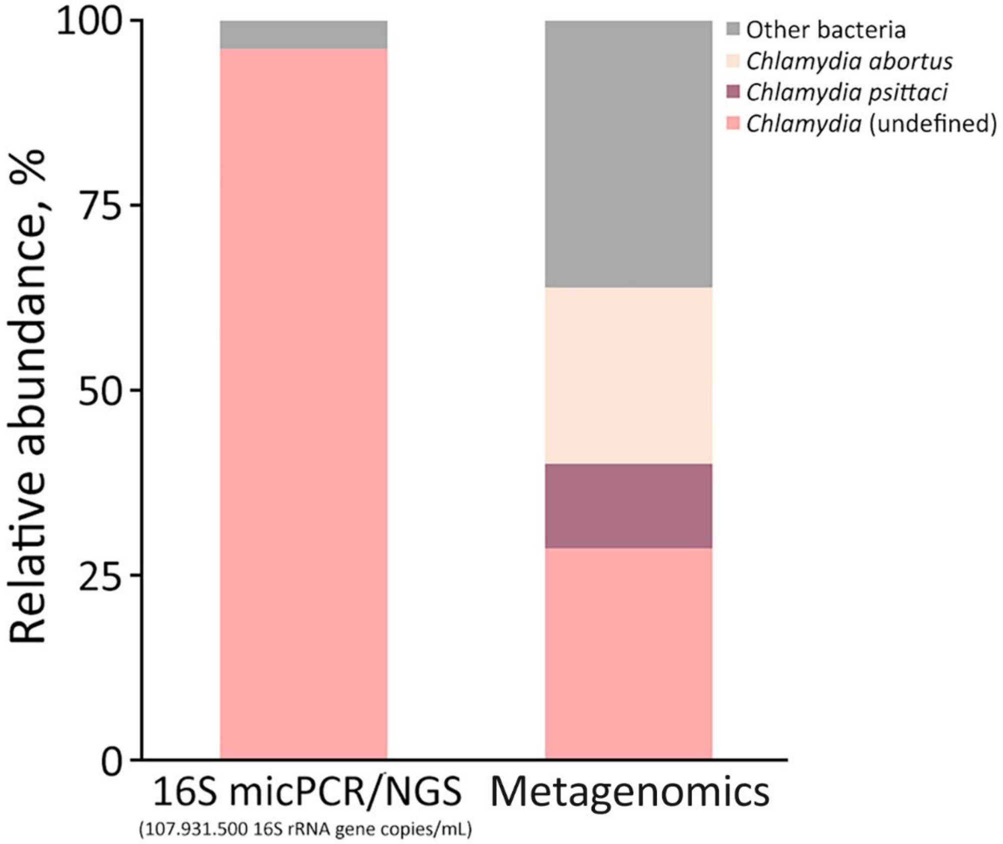

Appendix Figure 1. Bacterial microbiota profiles of the lower respiratory tract in a patient with community-acquired pneumonia caused by avian *Chlamydia abortus*, the Netherlands. A high relative abundance of *Chlamydia* spp. was detected in the lower respiratory tract by using quantitative 16S rRNA gene micelle PCR with next-generation sequencing analysis (micPCR/NGS) and shotgun metagenomics.

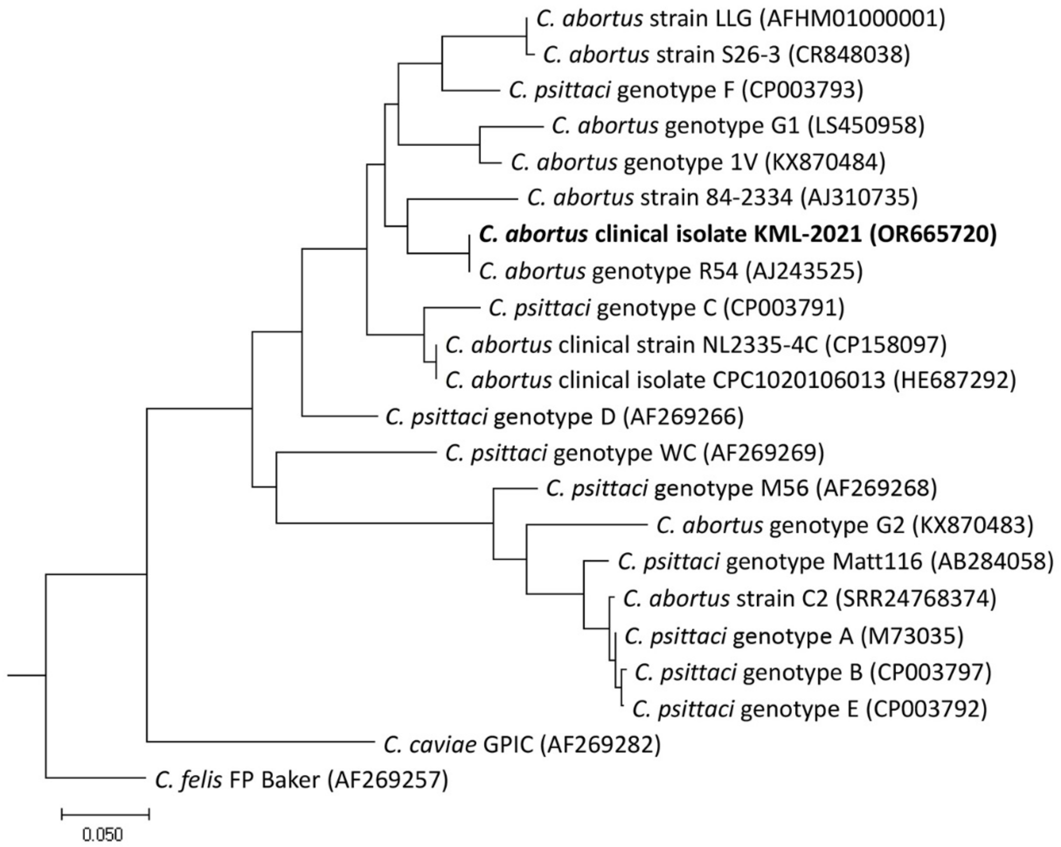

Appendix Figure 2. Chlamydial genotyping in a patient with community-acquired pneumonia caused by avian *Chlamydia abortus*, the Netherlands. For genotyping, an 850-bp alignment of *ompA* gene sequences from clinical isolate KML-2021 shown in bold (GenBank accession no. OR665720) and reference *Chlamydia* strains (GenBank accessions) was used. The tree was rooted with the *C. felis* (FP Baker) reference strain.
